# Retraction: Nanosized TiO_2_-Induced Reproductive System Dysfunction and Its Mechanism in Female Mice

**DOI:** 10.1371/journal.pone.0297255

**Published:** 2024-01-11

**Authors:** The PLOS ONE Editors

Following the publication of this article [1], multiple concerns were raised regarding the results and text. Specifically,

- Values described as standard error (SE) in Tables 1–5 consistently represent 5% of the mean.
- There is substantial text overlap between the Methods section [1] and other works by the same author group that were under consideration at the same time, including [2–4]. Text reuse was not cited or acknowledged in [1] as is required by PLOS policy.
- Error bars in Figures 1 and 2 do not appear uniform, and it is not clear if these error bars represent SE as described in the legends or if they were calculated as 5% of the mean as in the tables.
- The Methods section states that Student’s t-test was used to analyse the data, however given the number of groups and conditions in each experiment, this statistical test is not appropriate.
- Tables and figures contain letters described as indicating significant differences between groups, however it is not clear which groups are being compared.
- The numbers of animals reported in the Methods section and Results do not align.

The corresponding author stated that an error occurred in the calculation of SE values. They provided updated Tables 1–5 presenting mean ± standard deviation, as well as raw numerical data underlying the results presented in these tables.

In response to questions about the statistical analyses, the corresponding author stated that one-way ANOVA was used to compare groups in the tables. However, they did not clarify if a post-hoc test was used to compare individual groups, and their comments did not clarify the meaning of the letters in the tables or provide sufficient reporting of statistical analysis methods as needed to meet the journal’s requirements.

The corresponding author stated that text re-use occurred in the Methods section due to the similarity of the techniques with previous work from the group. Nevertheless, the original sources should have been cited in accordance with PLOS policy.

In light of the above issues, the article does not meet the journal’s reporting and policy requirements and PLOS remains concerned about the validity and reliability of the reported quantitative results. Therefore, the *PLOS ONE* Editors retract this article. We regret that the issues were not identified before the article was published.

All authors either did not respond directly to the editorial decision or could not be reached.
